# Supplementary material for: Laser Therapy in Heavily Treated Oncological Patients Improves Vaginal Health Parameters
Source: Cancers (Basel). 2024 Jul 31;16(15):2722. doi: 10.3390/cancers16152722 (PMC11311561; doi:10.3390/cancers16152722)
Supplement: Supplementary file 1 [file cancers-16-02722-s001.zip › cancers-3095168-supplementary.docx]

Supplementary Materials: Laser Therapy in Heavily Treated Oncological Patients Improves Vaginal Health Parameters

Marco Di Stanislao, Camelia Alexandra Coada, Francesca De Terlizzi, Stella Di Costanzo, Enrico Fiuzzi, Francesco Mezzapesa, Giulia Dondi, Damiano Paoli, Gianluca Raffaello Damiani, Francesco Raspagliesi, Giorgio Bogani, Antonino Ditto, Alessio Giuseppe Morganti, Pierandrea De Iaco and Anna Myriam Perrone

Supplementary methods

Evaluated parameters for vaginal health.

The tolerability and effects of laser therapy on vaginal health and sexual function was analyzed using the following parameters:

1 Visual analogue scale (VAS) [1]: the perceived pain intensity during laser therapy was assessed using a visual analogue score ranging from 0 to 10. A score of 0 indicated the absence of symptoms while a score of 10 represented the most severe symptoms.

2 Vaginal health index (VHI) is based on five parameters: vaginal elasticity, vaginal secretions, pH, epithelial mucous membrane, and vaginal hydration [2]. The final score defines the degree of atrophy in the genitourinary tract, and it is achieved by assigning a single score to each parameter.

3 Vaginal Length (VL) was measured using a graduated vaginal metal probe, which was also used for laser application. The average VL typically ranges from 7 to 10 centimeters and can be influenced by various factors such as age, hormonal status, body height and weight [3] as well as cancer treatments [4,5]. However, adequate values for VL are generally considered to fall within the range of 6 to 7cm [6]. For this study we considered the threshold ≥6cm.

4 Female Sexual Function Index (FSFI): patients were asked to complete a questionnaire on sexual function (Female Sexual Function Index - FSFI score) [7] which is composed by six domains for 19 questions; 17 of 19 include a 5-point scale and 2 of 19, a 6-point scale. The total score is obtained from the sum of the scores of all the domains and ranges from 2 to 36: desire/libido (2 questions), arousal (4 questions), vaginal lubrication (4 questions), orgasm (3 questions), global satisfaction or “global quality of life” (3 questions) and pain (3 questions). A total score >26.55 is commonly used as a cutoff score to distinguish women with normal sexual function from those with sexual dysfunction.

Patients grouping for statistical analyses

In the first part of the study, we analyzed the trend of the gynecological parameters in the entire cohort of patients. In a second phase, the study population was divided into three groups based on the number of cycles performed: 1 cycle (Group 1), 2 cycles (Group 2) or 3 cycles (Group 3), and then the gynecological parameters of the three groups were analyzed separately. Next, the response and evolution of the parameters were evaluated in the subgroups of patients receiving radiotherapy, hysterectomy and bilateral adnexectomy and chemotherapy/hormonal therapy. To evaluate the relationship between the time elapsed from the completion of radiotherapy-based treatments to the initiation of laser therapy and its effects, we categorized the patients into three subgroups: 5-12 months, 12-24 months, and over 24 months, based on the interval between the end of radiotherapy-based treatments and the start of laser therapy.

Supplementary Results

Sample size estimation

Given the lack of similar studies in the existing literature, we sought to establish the requisite minimum sample size for achieving the primary objective of this investigation *i.e.* the assessment of the impact of multiple laser treatment cycles on the vaginal health of oncological patients. We conducted a pilot study on the first 5 patients who completed 3 laser cycles. We considered the evolution of the vaginal health index across all treatment sessions and cycles and determined the repeated measures correlation coefficient, the effect size of the 3 cycles taking into account the means and standard deviation of the data at the beginning and at the end of the laser treatment. Thus, considering a statistical power (1−β) of 0.8 with and probability of type I error (alpha) of 0.05, we calculated that a minimum sample size of no less than 8 patients is required for this study.

**Table S1.** General characteristics of patients receiving laser cycles in this study. The patients were grouped based on the oncological treatment type they received prior to the laser treatment. N: number of patients; SD: standard deviation; BMI: body mass index; N/A: not available.

|  | | |  | **Patients group**  **N(%)/mean±SD** | | | | |  |
| --- | --- | --- | --- | --- | --- | --- | --- | --- | --- |
| **Variable** | | | **Total**  **N = 113** | **Radiotherapy and/or Brachytherapy**  **N = (65%)** | **Hysterectomy and Bilateral adnexectomy**  **N = (30%)** | | **Chemotherapy and anti-hormonal therapy**  **N = (19%)** | | **p-value** |
| **Age at oncological diagnosis** | | | 46.6±8.5 | 46.58+6.28 | 46.58+6.9 | | 48.03+9.32 | | 0.18 |
| **Age at first laser** | | | 50.5±8.3 | 51.11+6.79 | 51.11+6.42 | | 51.54+9.26 | | 0.39 |
| **Age group** | | **≤45** | 35(31) | 17(26.2) | 13(44.8) | | 5(26.3) | | 0.17 |
|  |  | **>45** | 78(69) | 48(73.8) | 16(55.2) | | 14(73.7) | |  |
| **Age at menopause** | | | 45.2±6.3 | 45.58+5.52 | 45.58+5.69 | | 46.11+6.6 | | 0.33 |
| **Early menopause <45years** | **No** | | 58(52.3) | 36(57.1) | 11(37.9) | | 11(57.9) | | 0.2 |
|  | **Yes** | | 53(47.7) | 27(42.9) | 18(62.1) | | 8(42.1) | |  |
|  | *N/A* | | *2* |  |  | |  | |  |
| **Premature menopause <40years** | **No** | | 94(84.7) | 54(85.7) | 23(79.3) | | 17(89.5) | | 0.57 |
|  | **Yes** | | 17(15.3) | 9(14.3) | 6(20.7) | | 2(10.5) | |  |
|  | *N/A* | | *2* |  |  | |  | |  |
| **BMI (kg/m2)** | | | 23.5±4.4 | 22.32+3.5 | 22.32+4.01 | | 23.93+4.77 | | 0.15 |
| **BMI category** | **Normal** | | 106(94.6) | 61(93.8) | 27(96.4) | 18(94.7) | | 0.88 | |
|  | **Overweight** | | 6(5.4) | 4(6.2) | 1(3.6) | 1(5.3) | |  |  |
| **Parity** | **Nulliparous** | | 43(39.1) | 23(35.9) | 13(48.1) | 7(36.8) | | <0.001 | |
|  | **Parous** | | 12(10.9) | 2(3.1) | 3(11.1) | 7(36.8) | |  |  |
|  | **Cesarean section** | | 55(50) | 39(60.9) | 11(40.7) | 5(26.3) | |  |  |
|  | *N/A* | | *3* |  |  |  | |  | |
| **Tumor type at diagnosis** | **Cervix** | | 42(37.2) | 40(61.5) | 2(6.9) | 0 | | <0.001 | |
|  | **Uterus** | | 30(26.5) | 18(27.7) | 12(41.4) | 0 | |  |  |
|  | **Rectal** | | 5(4.4) | 4(6.2) | 1(3.4) | 0 | |  |  |
|  | **Ovarian** | | 15(13.3) | 1(1.5) | 14(48.3) | 0 | |  |  |
|  | **Breast** | | 18(15.9) | 0 | 0 | 18(94.7) | |  |  |
|  | **Other*** | | 3(2.7) | 2(3.1) | 0 | 1(5.3) | |  |  |
| **Stage** | **I+II** | | 86(76.1) | 47(73.4) | 23(82.1) | 16(84.2) | |  | |
|  | **III+IV** | | 25(23.9) | 17(26.6) | 5(17.9) | 3(15.8) | |  |  |
|  | *N/A* | | *2* |  |  |  | |  | |
| **Number of laser cycles** | **I** | | 69(61.1) | 39(60) | 16(55.2) | 14(73.7) | | 0.36 | |
|  | **II** | | 31(27.4) | 16(24.6) | 11(37.9) | 4(21.1) | |  |  |
|  | **III** | | 13(11.5) | 10(15.4) | 2(6.9) | 1(5.3) | |  |  |

*other cancers include Bartholin tumors.

**Table S2.** Descriptive statistics showing the time intervals between lasers sessions and cycles.

| **Cycle** | **Interval** | **Time (months)**  **Median(P25;P75)** |
| --- | --- | --- |
| **I** | **T0-T1** | 1.4(1.16;1.98) |
|  | **T1-T2** | 1.4(1.17;2.33) |
|  | **T2-T3** | 1.17(1.17;1.63) |
| **I-II** | | 11.78(9.1;16.1) |
| **II** | **T0-T1** | 1.4(1.17;1.86) |
|  | **T1-T2** | 1.4(1.17;1.86) |
|  | **T2-T3** | 1.7(1.4;2.48) |
| **II-III** | | 15.4(12.1;19.6) |
| **III** | **T0-T1** | 1.63(1.35;2.22) |
|  | **T1-T2** | 1.4(1.17;2.1) |
|  | **T2-T3** | 1.17(1.17;1.63) |

**Table S3.** Descriptive statistics showing the overall evolution of the VHI, LV and FSFI parameters during the laser therapy sessions in all the study participants (related to Figure 2A).

| ***All study population*** | | | | | | | |
| --- | --- | --- | --- | --- | --- | --- | --- |
| **VHI (points)** | | | | **LV** (cm) | | **FSFI** (points) | |
| **Cycle number** | **Laser session** | **mean±SD** | **p-value** | **mean±SD** | **p-value** | **mean±SD** | **p-value** |
| **I**  N = 113 | **T0 Baseline** | 12.79±3.82 | **<0.001** | 6.36±1.87 | **<0.001** | 11.04±10.05 | **<0.001** |
|  | **T1** | 14.49±3.63 |  | 6.71±1.72 |  | 13.31±11 |  |
|  | **T2** | 15.34±3.74 |  | 6.72±1.67 |  | 15.7±10.66 |  |
|  | **T3** | 16.29±3.9 |  | 7.37±1.75 |  | 15.59±11.2 |  |
| **II**  N = 44 | **T0 Before cycle II** | 14.75±3.43 | **0.03** | 6.46±1.98 | **<0.001** | 14.71±9.95 | 0.48 |
|  | **T1** | 16.02±3.34 |  | 6.51±1.92 |  | 17.35±10.37 |  |
|  | **T2** | 16.78±3.41 |  | 6.48±2.18 |  | 14.36±9.15 |  |
|  | **T3** | 17.25±4.21 |  | 7.14±1.79 |  | 17.65±10.31 |  |
| **III**  N = 13 | **T0 Before cycle III** | 14.53±2.5 | **<0.001** | 6±1.77 | **0.006** | 11.71±8.83 | 0.31 |
|  | **T1** | 14.69±3.75 |  | 6±1.68 |  | 22.2±13.47 |  |
|  | **T2** | 16.23±3.58 |  | 6.07±1.7 |  | 3.65±2.33 |  |
|  | **T3** | 17.5±3.29 |  | 6.5±1.5 |  | 12.06±15.13 |  |

**Table S4.** Comparison of the percentage loss in VHI, LV, and FSFI after the first laser cycle, stratified by the timing of the second cycle initiation: Early (Within 18 Months) vs. Late (Beyond 18 Months) (related to Figure 4). SD: standard deviation.

| **%loss of previously gained effect** | **<18 months**  **mean±SD**  **N = 33** | **≥18 months**  **mean±SD**  **N = 11** | **p-value** |
| --- | --- | --- | --- |
| VHI (points) | 41+41.9 | 77.9+37.3 | **0.013** |
| LV (cm) | 34.7+47.1 | 49.2+48.4 | 0.38 |
| FSFI (points) | 33.4+43.5 | 66+42.9 | **0.036** |

**Figure S1.** Evolution of VHI, VL and FSFI in the patients’ groups. The patients were divided based on the number of laser cycles they received.

**
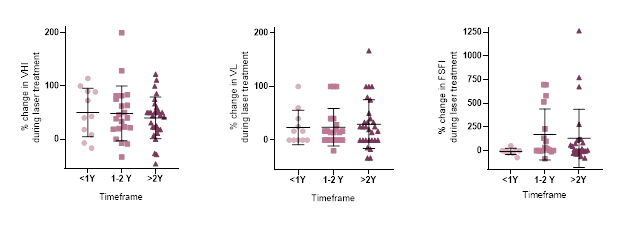
**

**Figure S2.** The impact of the time between oncological treatment completion and laser initiation on overall laser efficacy, stratified by the timeframe of the laser therapy initiation.

References

1. Heller, G.Z.; Manuguerra, M.; Chow, R. How to Analyze the Visual Analogue Scale: Myths, Truths and Clinical Relevance. *Scand. J. Pain* 2016, 13, 67–75. https://doi.org/10.1016/j.sjpain.2016.06.012.
2. Bachmann, G. Urogenital Ageing: An Old Problem Newly Recognized. Maturitas 1995, 22, S1–S5. https://doi.org/10.1016/0378-5122(95)00956-6.
3. Tan, J.S.; Lukacz, E.S.; Menefee, S.A.; Luber, K.M.; Albo, M.E.; Nager, C.W. Determinants of Vaginal Length. *Am. J. Obs. Gynecol*. 2006, 195, 1846–1850. https://doi.org/10.1016/j.ajog.2006.06.063.
4. Brand, A.H.; Bull, C.A.; Cakir, B. Vaginal Stenosis in Patients Treated with Radiotherapy for Carcinoma of the Cervix*. Int. J. Gynecol. Cancer* 2006, 16, 288–293. https://doi.org/10.1111/j.1525-1438.2006.00348.x.
5. Taha, O.T.; Al-Okda, N.; Hamdy, M.A. Vaginal length and sexual function after vertical versus horizontal closure of the vaginal cuff after abdominal hysterectomy: a randomised clinical trial. *J. Obstet. Gynaecol*. 2021, 42, 1–6, https://doi.org/10.1080/01443615.2021.1948512.
6. Dabaghi, S.; Zandi, M.; Ilkhani, M. Sexual Satisfaction in Patients with Mayer-Rokitansky-Küster-Hauser Syndrome after Surgical and Non-Surgical Techniques: A Systematic Review*. Int. Urogynecol. J*. 2019, 30, 353–362. https://doi.org/10.1007/s00192-018-3854-5.
7. Rosen, R.; Brown, C.; Heiman, J.; Leiblum, S.; Meston, C.; Shabsigh, R.; Ferguson, D.; D’Agostino, R. The Female Sexual Function Index (FSFI): A Multidimensional Self-Report Instrument for the Assessment of Female Sexual Function. *J. Sex Marital. Ther*. 2000, 26, 191–208. https://doi.org/10.1080/009262300278597.
